# The efficacy of anchored stand-alone spacers in comparison to conventional cage and plate in anterior cervical discectomy and fusion surgery: A meta-analysis of randomised controlled trials for clinical and radiological outcomes

**DOI:** 10.1016/j.bas.2024.102748

**Published:** 2024-01-18

**Authors:** Jake M. McDonnell, Salma Youssef, Tayler D. Ross, Harry Marland, Luke Turley, Gráinne Cunniffe, Stacey Darwish, Joseph S. Butler

**Affiliations:** a National Spinal Injuries Unit, Mater Misericordiae University Hospital, Dublin, Ireland; b Trinity Centre for Biomedical Engineering, Trinity Biomedical Sciences Institute, Trinity, Ireland; c School of Medicine, University of College Dublin, Belfield, Dublin, Ireland; d Orthopaedic Resident, University of Toronto, Canada; e School of Medicine, University of Galway, Galway, Ireland; f Department of Orthopaedics, Tallaght University Hospital, Tallaght, Dublin, Ireland; g Department of Orthopaedics, St. Vincent's University Hospital, Dublin, Ireland

**Keywords:** Cervical spine, ACDF, Spine surgery, Cage, Spacer, Outcomes

## Abstract

**Introduction:**

Anterior cervical discectomy and fusion (ACDF) is commonly performed with cage and plate constructs to stabilise diseased or injured cervical segments. Despite it being a commonly performed procedure, there are notable rates of associated morbidity reported in the literature. Stand-alone spacers represent a novel form of instrumentation to conventional cage and plate constructs.

**Research question:**

Do stand-alone spacers have improved operative characteristics and postoperative outcomes in ACDF cohorts when compared to cage and plate constructs?

**Methods:**

A systematic review and meta-analysis was conducted of PubMed/Medline, Embase and Google Scholar databases per the *Preferred Reporting Items for Systematic Reviews and Meta-Analyzes* guidelines. Studies of interest included cage and plate instrumentation versus anchored stand-alone spacers for patients undergoing ACDF. Pre- and post-operative clinical and radiological outcomes were collated and compared for significance between cohorts.

**Results:**

10 RCTs were identified and included with 779 patients total. Mean age of the entire cohort was 50.1 years. 62% (483/779) of the cohort were male. 384 patients underwent ACDF with stand-alone cage, while 395 had ACDF with conventional cage and plate. Stand-alone spacers significantly outperformed conventional instrumentation in terms of estimated blood loss (p < 0.01), total postoperative complications (p < 0.01), dysphagia rates (p = 0.04) and adjacent segment disease (p = 0.04). Non-inferiority was evident in both patient reported outcome measures and radiological outcomes.

**Conclusion:**

This meta-analysis highlights the efficacy of stand-alone spacers for the management of primarily cervical spondylitic disease for both single-level and multi-level pathology, and thus presents an attractive alternative to conventional instrumentation for patients undergoing ACDF surgery.

## Highlights

• Cage and plate constructs have notable rates of associated morbidity with ACDF.
• Stand-alone spacers are a novel form of instrumentation to be used in ACDF.
• Stand-alone spacers are an attractive alternative to cage and plate constructs.

## 1 Introduction

Anterior cervical fusion was first performed in 1955 by Robinson and Smith, who published a descriptive case series of their first eight patients undergoing the novel approach at the time (Robinson, 1955). In 1962, they described a more comprehensive cohort of 146 patients who underwent anterior interbody fusion of the cervical spine (Robinson et al., 1962). Fusion rates at follow-up were reported as 88%, with 89.6% of patients reporting satisfactory postoperative patient reported outcome measures (PROMs). There were no cases of mortality noted, with only 10 cases of “temporary” complications, all which occurred in the first 14 patients while still developing their experience and perfecting the technique. In the modern era of spine surgery, the majority of anterior cervical surgeries are associated with the implantation of instrumentation to stabilise the cervical level of injury. Anterior discectomy and fusion (ACDF) is typically performed with implementation of screws with either a cage and/or plate for further stabilisation (Fountas et al., 2007).

**Fig. 1:**
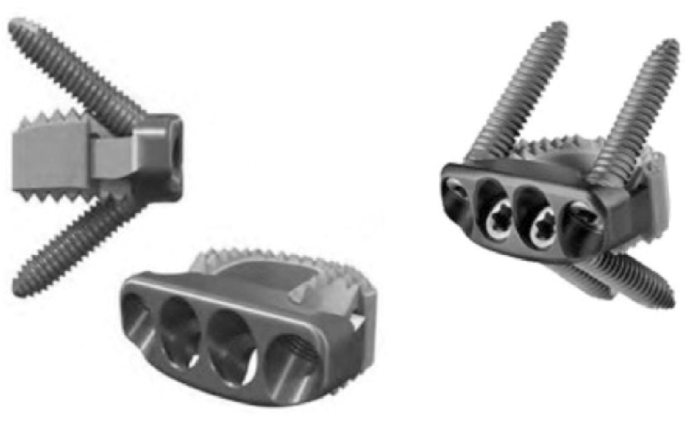
Schematic Design of Zero-P® Implant (Synthes GmbH, Oberdorf, Switzerland). Adapted from Son et al.^4^ 2014 under the under the Creative Commons Attribution-Non-Commercial License 4.0 (CCBY-NC).

Although several decades have passed since its inception, there remains a notable risk of procedure related morbidity with ACDF, leading to prolonged hospital stays and necessary intervention from allied health colleagues such as Speech and Language Therapy (SLT) (Fountas et al., 2007). In order to reduce procedure related complications, there has been a desire to improve surgical techniques and instrumentation for anterior cervical surgeries. As a result, anchored stand-alone spacers were developed for use in ACDF (Fig. 1) cohorts (Son et al., 2014; Sommaruga et al., 2021).

**Fig. 2:**
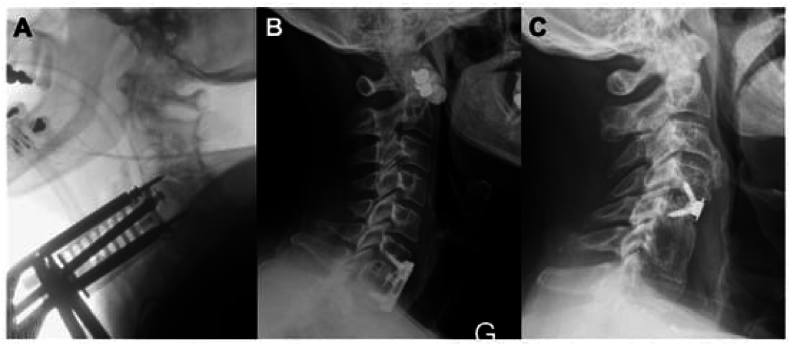
Anterior cervical cage placement and fixation. (A) Intraoperative lateral X-ray to assess ACDF cage placement, with Casper pins shown. (B) Post-operative lateral X-ray showing a single level ACDF at C6–C7 secured by a plate (Venture, Medtronic). (C) Post-operative lateral X-ray showing a single level ACDF at C4-5 (Zero-P® cage, DePuy Synthes). Adapted from Sommaruga et al.^5^ 2021 under the under the Creative Commons Attribution-Non-Commercial License 4.0 (CCBY-NC).

These spacers are designed to provide stabilisation at the injured level without the need of a plate and subsequently reducing operative time, estimated blood loss (EBL) and the amount soft tissue dissection. While several commercial models are available, no consensus exists regarding the efficacy of novel stand-alone spacers in comparison to conventional ACDF instrumentation (Fig. 2). That serves the premise for this systematic review and meta-analysis, in order to provide practicing spine surgeons with the evidence necessary for informed decision making and appropriate choice of implant when performing anterior cervical discectomy and fusion.

## 2 Methods

### 2.1 Search strategy and study selection

Two independent reviewers (JMM and LT) performed a literature search following the *Preferred Reporting Items for Systematic Reviews and Meta-Analyzes (PRISMA)* guidelines (Liberati et al., 2009). Any disagreements regarding study inclusion were resolved by consulting the opinion of a senior author (SD or JSB). A comprehensive search was performed for eligible articles using the PubMed/Medline, Embase, and Cochrane databases to include studies up to, and including July 10th, 2022 (PROSPERO ID: 353,719). Search terms included “anterior cervical surgery” AND (“randomised controlled trial” OR “randomised controlled trial” OR “RCT” OR “trial” OR “random”) AND (“spacer” OR “plate” OR “screw” OR “cage”) AND (“outcomes” OR “complications” OR “dysphagia” OR “pain” OR “function”) AND (“follow-up”). Reference lists of full-text articles were reviewed and screened for further studies meeting the inclusion criteria.

### 2.2 Eligibility criteria

The inclusion criteria were (i) randomised controlled studies pertaining to (ii) anterior cervical surgery of patients (iii) managed with an anchored spacer and conventional instrumentation (plates, screws, cages) (iii) that reported comparative outcomes and (iv) were written in English/had translated version available. The exclusion criteria included (i) non-randomised studies (ii) non-comparative outcomes (case series or case reports).

### 2.3 Data extraction

All relevant information was collected by two independent reviewers. The *Methodological Quality of Evidence (MQOE)* was assessed using the Risk of Bias 2 (RoB-2) tool developed by Cochrane for evaluating bias in randomised studies (Sterne et al., 2019).

### 2.4 Outcomes analysed and statistics

Outcomes analysed were operative characteristics (operative time, EBL, etc), postoperative complications, and PROMS. All statistical analysis was performed using The R Project for Statistical Computing (version 4.1.2). Heterogeneity between studies was quantified using the I^2^ statistic. A random effects model and binary outcomes model were employed. Results are expressed as mean for continuous outcomes and risk ratio (RR) for dichotomous outcomes, with a 95% confidence interval (CI). Heterogeneity values were interpreted per Cochrane values; (i) 0%–40% = low degree of heterogeneity (ii) 30%–60% = moderate degree of heterogeneity (iii) 50%–90% = substantial degree of heterogeneity (iv) 75%–100% = considerable degree of heterogeneity. A p-value of <0.05 was considered statistically significant.

### 2.5 Aims and objectives

The aim of this study was to compare anchored spacers and conventional instrumentation employed in anterior cervical spine surgery in terms of operative characteristics and postoperative outcomes.

The objective of this study is to elucidate if anchored spacers have improved operative characteristics and postoperative outcomes and can be inferred as efficacious as conventional instrumentation in the management of cervical spine disorders.

## 3 Results

**Fig. 3:**
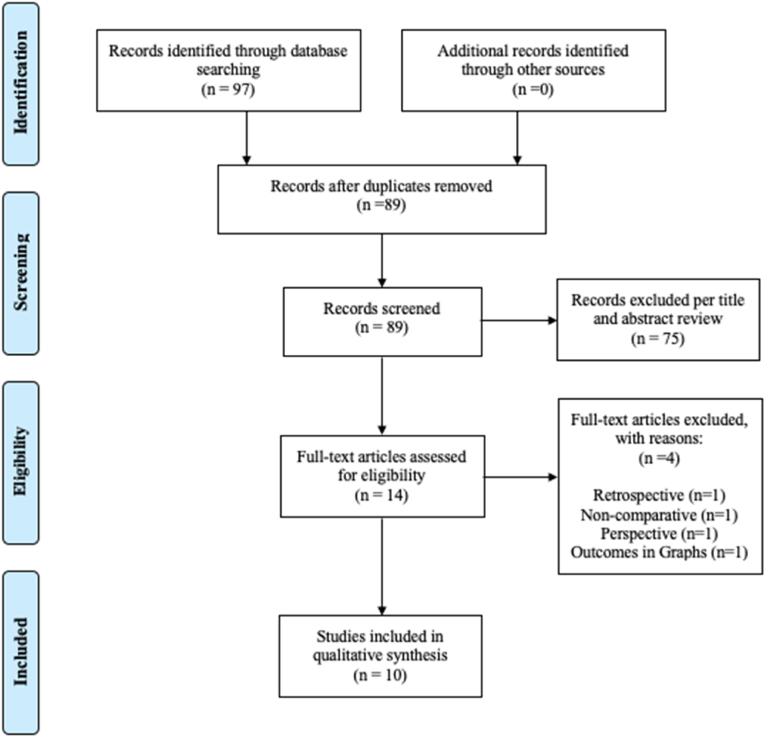
PRIMSA flowchart per the “Preferred Reporting Items for Systematic Reviews and Meta-Analyzes” (PRISMA) guidelines.

The literature search yielded 97 results (Fig. 3). After removal of duplicates, 89 articles remained for screening of title and abstract. 75 articles were subsequently excluded, leaving 14 for full-text review. 11 studies met the predefined inclusion criteria and were included for full text review. One text was further excluded due to inability to extract sufficient data as results were presented in graph format. Thus, 10 studies were included in for qualitative synthesis of the literature and meta-analysis (Nemoto et al., 2015; Yan and Nie, 2015; Chen et al., 2016; Liu et al., 2016; Li et al., 2017; Panchal et al., 2017; Xiao et al., 2017; He et al., 2018; Scholz et al., 2020; Zavras et al., 2022).

Overall, there were 779 patients included across the 10 studies. Mean age of entire cohort was 50.1 years (range 30–78 years). Sex distribution was 62% (483/779) male and 38% (296/779) female. 384 patients underwent ACDF with stand-alone cage, while 395 had ACDF with conventional instrumentation (cage and plate). Most studies defined cohort characteristics inclusive of a diagnosis of cervical spondylitic radiculopathy or myelopathy with failed non-operative management. The characteristics of studies are outlined in Table 1.

**Table 1:** Characteristics of studies.

Author	Year	Cohort Characteristics	Sample Size	Mean Age (Range)	Spacer: Cage & Plate	Male: Female	Single-level or Multi-level	Follow-up
Nemoto et al.^8^	2015	Cervical spondylitic radiculopathy with failed trial period of conservative therapy (non-specified)	46	41.2 (31–54)	24:22	42:4	Single-level	2-years
Yan et al.^9^	2015	Cervical spondylitic associated radiculopathy or myelopathy	98	43.2 (NR)	49:49	58:40	Single-level and Multi-level	6-months
Chen et al.^10^	2016	Cervical spondylitic myelopathy with failed trial period of conservative therapy of 1-year	72	52.9 (NR)	34:38	46:26	Multi-level	3-years
Liu et al.^11^	2016	Cervical spondylitic myelopathy	60	57.1 (NR)	28:32	22:38	Multi-level	Follow-up range (12–36 months
Li et al.^12^	2017	Cervical spondylitic radiculopathy with failed trial period of conservative therapy of 6-weeks	138	51 (30–69)	68:70	86:52	Single-level and Multi-level	>2 years
Panchal et al.^13^	2017	Cervical disc disease requiring ACDF at 1 intervertebral level with failed trial period of conservative therapy for at least 6-weeks	54	48.8 (NR)	26:28	25:29	Single-level	Average follow up 11.0 ± 2.5 months for the Stand-alone group and 10.6 ± 2.8 months for the Plate group.
Xiao et al.^14^	2017	Cervical spondylopathy ± radiculopathy/myelopathy	120	43.0 (NR)	60:60	68:52	Single-level	up to 6-months
He et al.^15^	2018	Cervical spondylotic myelopathy requiring multi-level ACDF with failed trial period of conservative therapy of at least six months	104	57.5 (40–78)	52:52	55:49	Multi-level	>2-years
Scholz et al.^16^	2020	Cervical spondylitic radiculopathy with failed trial period of conservative therapy (non-specified)	41	54.0 (36-76	21:20	24:17	Multi-level	2-years
Zavras et al.^17^	2022	Sub-axial cervical spine of various aetiologies with failed trial period of conservative therapy (non-specified period)	46	57.5 (NR)	22:24	30:16	Single-level and Multi-level	1-year

(NR) = not reported.

Stand-alone cages were reported favourable in some capacity in 4/10 studies. One study favoured conventional instrumentation for short-term stability and 5/10 studies reported equivocal outcomes. Comparative instrumentation and conclusions of respective studies are outlined in Table 2.

**Table 2:** Comparative instrumentation and conclusion among respective studies.

Author	Year	Stand-Alone Spacer	Conventional Instrumentation	Conclusion
Nemoto et al.	2015	PREVAIL® (Medtronic Sofamor Danek, Memphis, TN, USA).	PEEK cage (CORNERSTONE©, Medtronic Sofamor Danek, Memphis, TN) + plate (PREMIER©, Medtronic Sofamor Danek, Memphis, TN, USA)	Stand-alone spacer significant lower rates of ASD. Non-inferior/Equivocal with regards to other outcomes
Yan et al.	2015	Zero-P® (Johnson & Johnson, New Brunswick, New Jersey, USA)	“anterior cervical plate interbody fusion system” (non-specified)	Stand-alone spacer cohort associated with significantly less rates of dysphagia, and improved PROMS
Chen et al.	2016	Zero-P® (Depuy Syncage; Depuy Synthes, USA)	PEEK cage (Solis©, Stryker, USA) + plate (Atlantic, Medtronic, Sofamor, USA)	Stand-alone spacers non-inferior/Equivocal
Liu et al.	2016	Zero-P® (LDR, Troyes, France).	PEEK cage + plate (Medtronic, Minneapolis, MN, USA)	Stand-alone spacers significantly improved clinical outcomes (operative time, EBL, complications) Equivocal with regards radiological outcomes
Li et al.	2017	Fidji® (Abbott Spine, Bordeaux, France)	PEEK cage + plate (SLIMLOC© or SKYLINE©) (Both DePuy Spine, Johnson & Johnson, New Brunswick, NJ, USA)	Stand-alone spacers non-inferior/Equivocal
Panchal et al.	2017	COALITION® Spacer (Globus Medical, Inc., Audubon, Pennsylvania, USA)	Spacer (COLONIAL©) + Plate (PROVIDENCE©) (Both Globus Medical, Inc., Audubon, Pennsylvania, USA)	Stand-alone spacers non-inferior/Equivocal
Xiao et al.	2017	Zero-P® (Johnson & Johnson, New Brunswick, New Jersey, USA)	Cage + plate (PCB©, Synthes, Swiss)	Stand-alone spacer cohort associated with significantly improved clinical outcomes
He et al.	2018	Zero-P® (Synthes GmbH Switzerland, Oberdorf, Switzerland)	“Standard ACDF with anterior cervical plate” (non-specified)	Stand-alone spacers had non-inferior PROMS and reduced (non-significant) complication rates
Scholz et al.	2020	Zero-P® (Synthes GmbH Switzerland, Oberdorf, Switzerland)	Cage (Syncage-C©) + Plate (Cervical Spine Locking Plate©) (Both Synthes GmbH Switzerland, Oberdorf, Switzerland)	Stand-alone spacers non-inferior/Equivocal
Zavras et al.	2022	“interbody cage with an integrated 3-screw construct” (non-specified)	“interbody device and anterior plating” (non-specified)	Equivocal. Conventional instrumentation may provide more stability in immediate postoperative period.

**Fig. 4:**
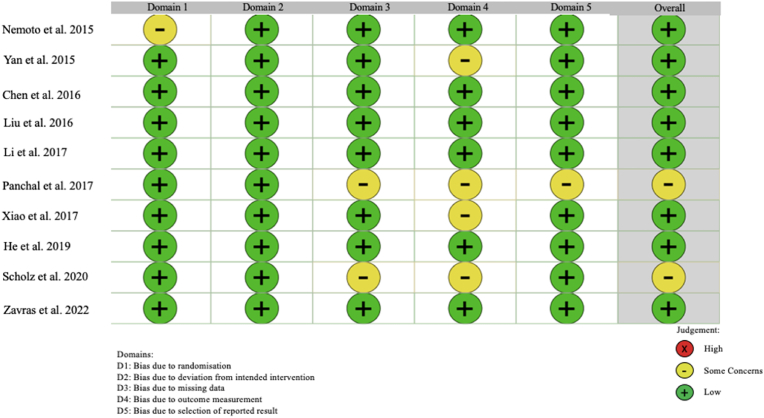
Risk Of Bias (ROB) 2 Cochrane tool for evaluating bias in randomised controlled studies.

Risk of bias was evaluated using the RoB-2 Cochrane tool, as outlined in Fig. 4. Overall, 2 studies had some concerns with regards to their methodology, while 8 studies convey low concerns. Of the 10 studies 2/10 reported industry finance or relevant conflicts (Panchal et al., 2017; Scholz et al., 2020), 6/10 reported no industry finance or relevant conflicts, and 2/10 did not list author conflicts of any nature (Chen et al., 2016; Xiao et al., 2017).

i. Operative Characteristics
  a Operative Time

**Fig. 5:**
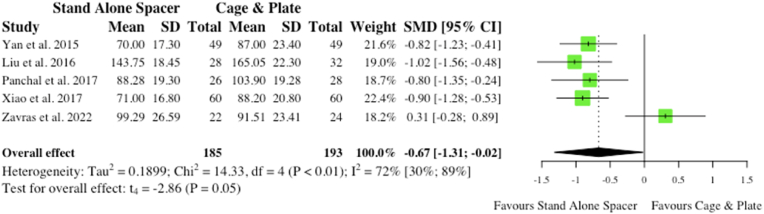
Comparative operative time (in minutes) for cohorts undergoing ACDF with anchored stand-alone spacer versus conventional cage and plate. (SMD) = standardised mean difference. (95% CI) = 95% confidence interval. (SD) = standard deviation.

Five studies reported on operative time for both stand-alone cage and conventional instrumentation cohorts. Two studies were concerned with only single-level surgery, while one study included only multi-level surgery and two studies were inclusive of both single- and multi-level surgery. Overall, operative time was notably shorter with stand-alone spacer placement (SMD: 0.67; 95% CI: 1.31,-0.02; p = 0.05) which depicted borderline significance, as outlined in Fig. 5.

b. Estimated Blood Loss

**Fig. 6:**
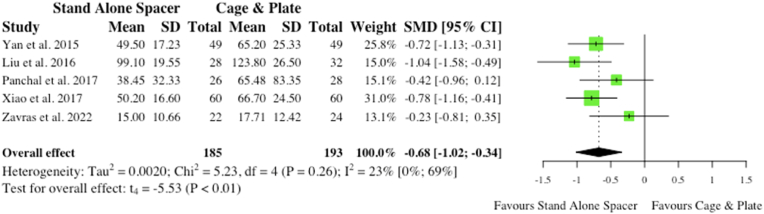
Comparative estimated blood loss (in millilitres) for cohorts undergoing ACDF with anchored stand-alone spacer versus conventional cage and plate. (SMD) = standardised mean difference. (95% CI) = 95% confidence interval. (SD) = standard deviation.

Five studies reported on mean estimated blood loss (EBL) for respective cohorts. Similarly, two studies related to single-level surgery only, while another study concerned multi-level surgery and one study was inclusive of both. Overall, stand-alone spacer placement was associated with a significantly lower EBL when compared to conventional instrumentation (SMD: 0.68; 95% CI: 1.02, −0.34; p < 0.01), as shown in Fig. 6. However, this likely has no significance clinically as blood loss was <100 ml in all cohorts but one.

ii. Postoperative Complications
  a. Total Complications (Inclusive of Dysphagia and ASD)

**Fig. 7:**
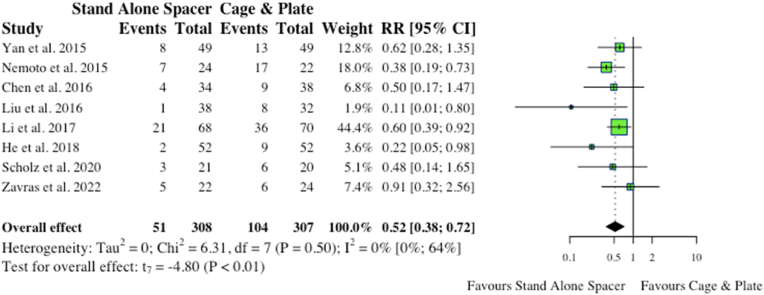
Comparative total postoperative complications (inclusive of dysphagia and ASD) for cohorts undergoing ACDF with anchored stand-alone spacer versus conventional cage and plate. (RR) = relative risk. (95% CI) = 95% confidence interval.

Eight studies reported on total complications in the acute postoperative period, inclusive of dysphagia and adjacent segment disease (ASD). Interestingly, anterior cervical surgery with anchored spacers was associated with significantly fewer postoperative complications (51/308, 16.6% vs 104/307, 33.9%; RR: 0.52; p < 0.01) compared to conventional cage and plate (Fig. 7).

b. Total Complications (Exclusive of Dysphagia and ASD)

**Fig. 8:**
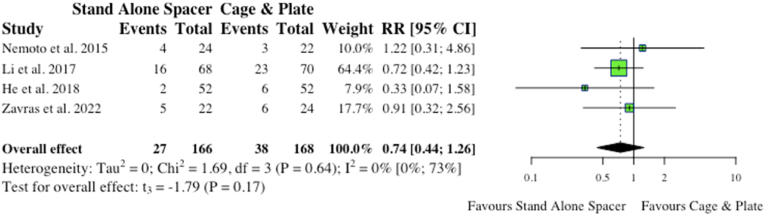
Comparative total postoperative complications (exclusive of dysphagia and ASD) for cohorts undergoing ACDF with anchored stand-alone spacer versus conventional cage and plate. (RR) = relative risk. (95% CI) = 95% confidence interval.

When omitting dysphagia and ASD, two major complications associated with anterior cervical surgery, four studies reported on other postoperative complications inclusive of epidural haematoma, respiratory infections, wound infections, etc. Comparatively, stand-alone spacers had a lower amount of postoperative complications (27/166, 16.3% vs 38/168, 22.6%; RR:0.74) versus conventional instrumentation, however this did not reach statistical significance (p = 0.15). The results are depicted in the forest-plot shown in Fig. 8.

c. Dysphagia

**Fig. 9:**
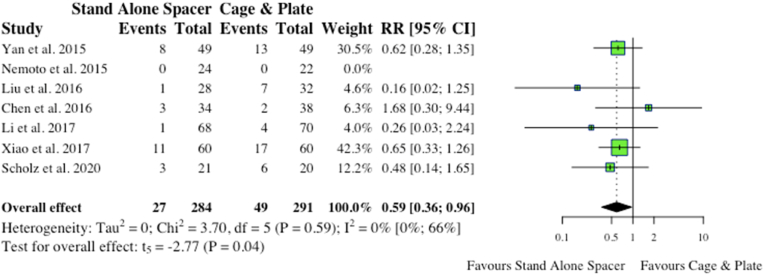
Comparative rates of dysphagia at 3-months for cohorts undergoing ACDF with anchored stand-alone spacer versus conventional cage and plate. (RR) = relative risk. (95% CI) = 95% confidence interval.

Overall, seven studies reported on persistent dysphagia post-op. Four studies reported on dysphagia at a specified 3-month follow-up period from date of operation. Three of these four studies, along with another four studies, further outlined dysphagia at “final follow-up” or “follow-up” greater than 3-months. All studies used Bazaz model of for incidence and grading of dysphagia, while Chen et al. (2016) also report using short Swallowing and Quality of Life (SQOL) questionnaires. In regards to postoperative dysphagia at three months, no significance existed between stand-along spacer and conventional instrumentation (17/143, 11.9% vs 24/142, 16.9%; RR: 0.65; p = 0.62). However, with regards to dysphagia > 3-months, the anchored spacer cohort collectively had fewer patients complaining of prolonged dysphagia (>3-months) compared to conventional instrumentation (27/284, 9.5% vs 49/291, 21.2%; RR: 0.59). This proved statistically significant on meta-analysis (p = 0.04), as highlighted in Fig. 9.

No meaningful collective analysis could be performed for post-op severity of dysphagia. Three studies (Nemoto et al. (2015), Yan et al. (Yan and Nie, 2015) and Liu et al. (2016)) only report incidence of dysphagia, while the remaining four studies (Chen et al. (2016), Li et al. (2017), Xiao et al. (2017) and Scholz et al. (2020)) report on post-op severity of dysphagia. Chen et al. (2016) mentioned using SQOL for post-op severity, reporting no significant difference between the stand-alone spacer and control group on pre-op (p = 0.80) and post op (p = 0.48) scores. Comparatively, Li et al. (2017) discusses the post-op severity of dysphagia in the main-text, noting that 3 patients (2 in stand-alone spacer, 1 in control group) had residual, mild dysphagia at 3-months post-op. It is also mentioned that one patient (not specified which group) had moderate dysphagia, while another had severe dysphagia post-op (control group), however this was due to food aspiration. Xiao et al. (2017) report that in the experimental stand-alone spacer group, there were fewer reports of moderate dysphagia (3/11, 27%) compared to the control cage and plate group (5/17, 29.4%). Although significance is reported, this is in relation to the incidence of dysphagia and not severity of dysphagia. Scholz et al. (2020) noted in the main-text that as complaints of dysphagia as follow-up were classified as mild and that no patient complained of moderate or severe dysphagia.

d. Adjacent Segment Disease

**Fig. 10:**
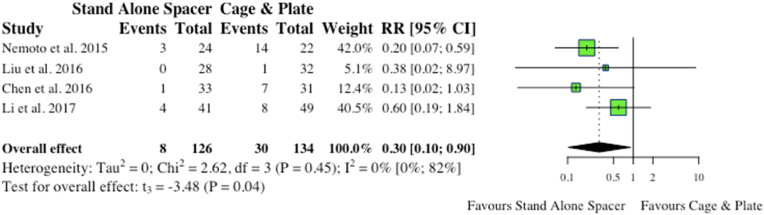
Comparative rates of dysphagia for cohorts undergoing ACDF with anchored stand-alone spacer versus conventional cage and plate. (RR) = relative risk. (95% CI) = 95% confidence interval.

Four studies reported on ASD in the postoperative period. Two vaguely defined it as adjacent segment disease, while two studies specified adjacent segment ossification. Overall, stand-alone spacer patients were found to have significantly less postoperative occurrence of ASD compared to the conventional instrumentation cohort (8/126, 6.4% vs 30/134, 22.4%; RR: 0.30; p = 0.04), as shown in Fig. 10.

iii. Radiological Outcomes
  a. Disc Height

**Fig. 11:**
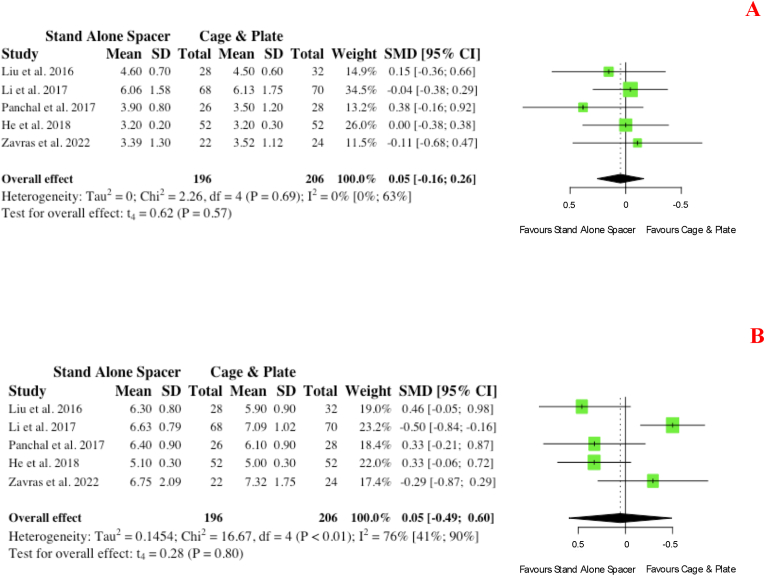
Comparative (A) pre-operative and (B) postoperative intervertebral disc height (in millimetres) for cohorts undergoing ACDF with anchored stand-alone spacer versus conventional cage and plate. (SMD) = standardised mean difference. (95% CI) = 95% confidence interval. (SD) = standard deviation.

Five studies report on comparative pre- and postoperative disc height between cohorts. Pre-operatively, no significance existed (p = 0.57) between cohorts (Fig. 11 A). Similarly, in the post-operative period, no significance existed between those undergoing anterior cervical surgery with stand-alone spacers or conventional instrumentation (SMD: 0.05; 95% CI: 0.49,0.60; p = 0.80) (Fig. 11 B).

b. Cervical Lordosis

**Fig. 12:**
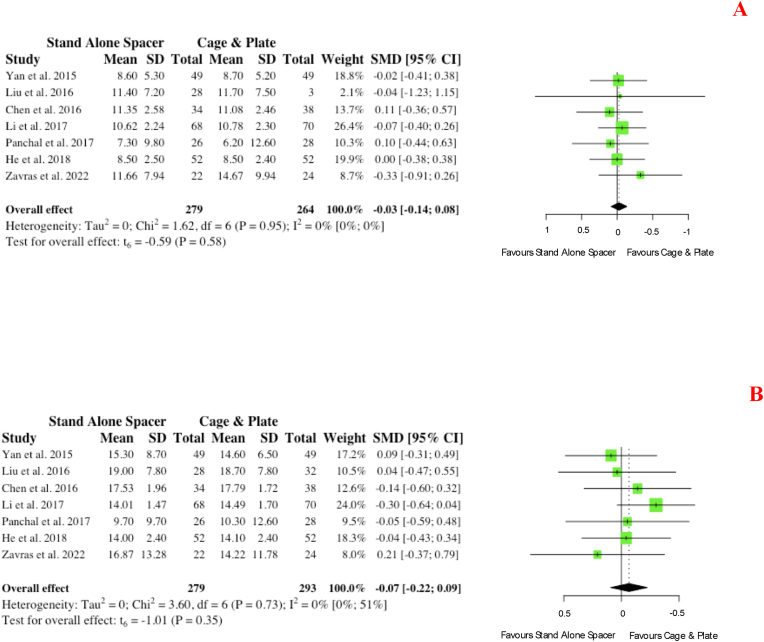
Comparative (A) pre-operative and (B) postoperative cervical lordosis (in degrees) for cohorts undergoing ACDF with anchored stand-alone spacer versus conventional cage and plate. (SMD) = standardised mean difference. (95% CI) = 95% confidence interval. (SD) = standard deviation.

Overall, seven studies reported on pre- and post-operative measurements of cervical lordosis for groups. Pre-operatively, no significance (p = 0.58) existed (Fig. 12 A). Similarly, on post-operative measurements at follow-up, no significance existed for those who received stand-alone spacer placement versus conventional cage and plate (SMD: 0.07; 95% CI: 0.22,0.09; p = 0.35), as depicted in Fig. 12 B.

iv. Patient Reported Functional Outcomes
  a. Neck Disability Index

**Fig. 13:**
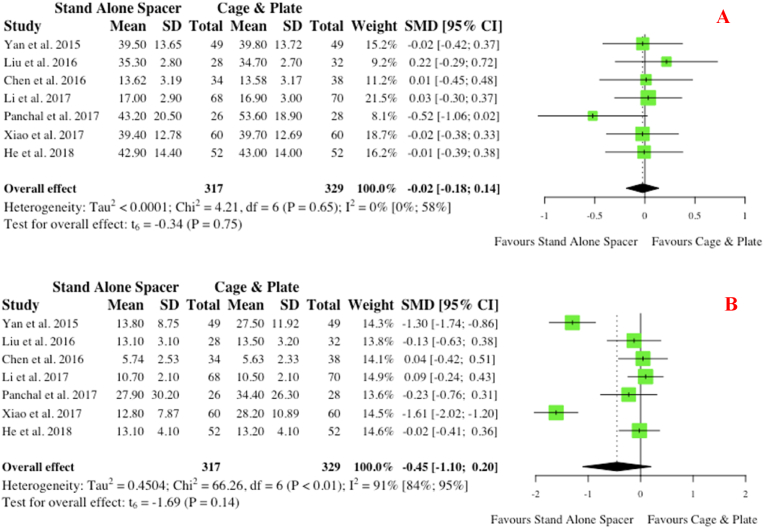
Comparative (A) pre-operative and (B) postoperative neck disability index scores for cohorts undergoing ACDF with anchored stand-alone spacer versus conventional cage and plate. (SMD) = standardised mean difference. (95% CI) = 95% confidence interval. (SD) = standard deviation.

Seven studies report pre- and post-operative patient reported outcome measures with relation to the validated and widely implemented Neck Disability Index (NDI). No difference in pre-operative NDI scores was noted between groups (p = 0.75). A trend towards improved post-operative NDI scores was evident (Fig. 13 B) for the anchored spacer group (SMD: 0.45; 95% CI -1.10,0.20), however this did not reach statistical significance (p = 0.14).

b. Japanese Orthopaedic Association Score

**Fig. 14:**
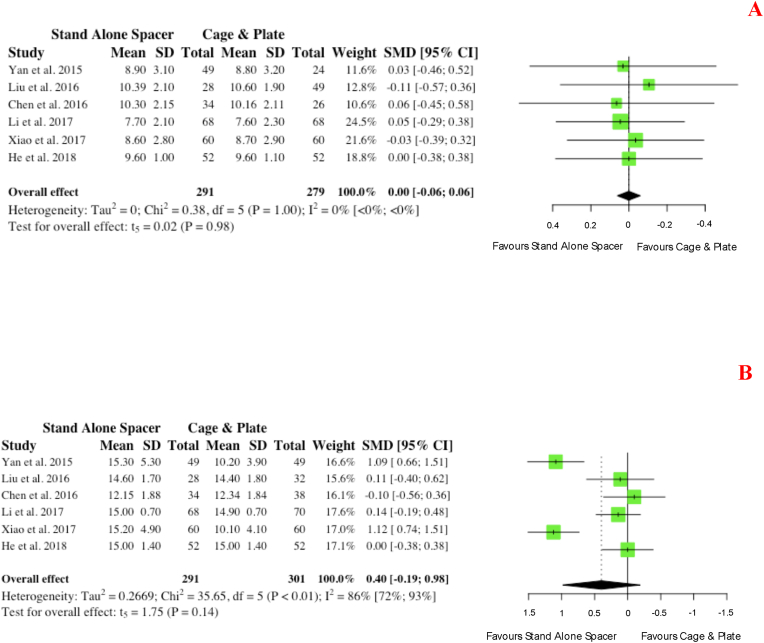
Comparative (A) pre-operative and (B) postoperative Japanese orthopaedic association scores for cohorts undergoing ACDF with anchored stand-alone spacer versus conventional cage and plate. (SMD) = standardised mean difference. (95% CI) = 95% confidence interval. (SD) = standard deviation.

Six studies reported on Japanese Orthopaedic Association (JOA) score for functional status. Similarly to the NDI, no pre-operative differences were noted between cohorts (p = 0.98), with a non-significant trend of improved post-operative JOA scores seen on meta-analysis for the anchored spacer cohort compared to conventional instrumentation (SMD: 0.40; 95% CI: 0.19,0.98; p = 0.14), as shown in Fig. 14.

c. Visual Analogue Score

**Fig. 15:**
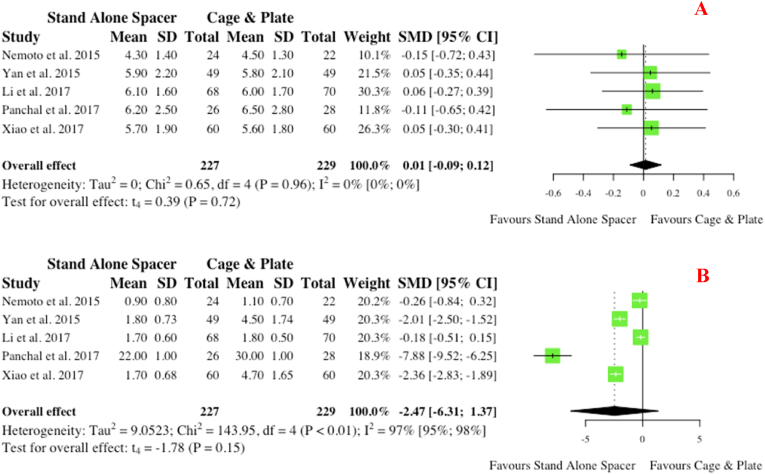
Comparative (A) pre-operative and (B) postoperative visual analogue scale scores for cohorts undergoing ACDF with anchored stand-alone spacer versus conventional cage and plate. (SMD) = standardised mean difference. (95% CI) = 95% confidence interval. (SD) = standard deviation.

Five studies outline pre- and post-operative Visual Analogue Scale (VAS) scores for neck pain. Comparably to NDI and JOA scores, no pre-operative differences in VAS scores were noted (p = 0.72), with a trend towards improved VAS scores for the anchored spacer group (SMD: 2.47; 95% CI: 6.31,1.37) which did not reach statistical significance (p = 0.15) (Fig. 15).

v. Fusion Rates

**Fig. 16:**
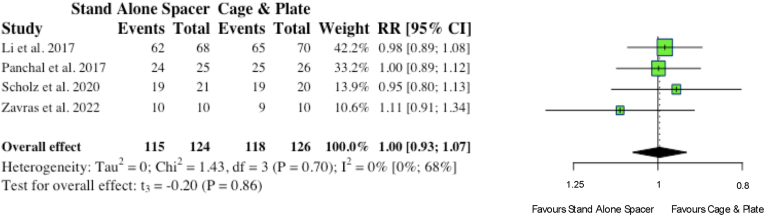
Comparative fusion rates at follow-up for cohorts undergoing ACDF with anchored stand-alone spacer versus conventional cage and plate. (RR) = relative risk. (95% CI) = 95% confidence interval.

Four studies reported on fusion rates at final follow-up. Collectively, no significance existed between anchored spacers and conventional cage and plate in terms of achieving fusion in patients undergoing anterior cervical surgery (115/124, 92.7% vs 118/126, 93.7%; p = 0.86), as highlighted in Fig. 16.

vi. Multi-level Pathology

Sufficient studies (≥3 studies) to run meaningful statistical analysis reported on EBL, total operative time, total postoperative complications (inclusive of dysphagia and ASD), dysphagia, ASD and cervical lordosis for patients undergoing multi-level anterior cervical spine surgery managed with either anchored spacer or conventional instrumentation.

a. Operative Characteristics

Four studies reported on EBL, with the anchored spacer cohort associated with a significantly reduced amount of EBL (SMD: 0.66; 95% CI: 1.11,-0.21; p = 0.02) and operative time (SMD: 0.76; 95% CI: 1.21,-0.30; p = 0.01) when compared to the conventional instrumentation group.

b. Postoperative Complications

Four studies reported on total complications, inclusive of dysphagia and ASD. Overall, the anchored spacer group had fewer complications (10/115, 8.7% vs 25/114, 21.9%), although this did not reach significance (RR:0.45; 95% CI: 0.15,1.39; p = 0.11). When concerned with only dysphagia and ASD, three studies reported on prolonged dysphagia (>3 months) and three studies on ASD respectively. No significance existed between cohorts in terms of postoperative rates of dysphagia (7/83, 8.4% vs 15/90, 16.7%; RR:0.54; 95% CI: 0.04,7.60; p = 0.42) or ASD (2/83, 2.4% vs 8/87, 9.2%; RR:0.40: 95% CI 0.01,21.99; p = 0.43) for patients undergoing multi-level anterior cervical surgery.

c. Radiological Outcomes

Three studies reported on cervical lordosis measurements at follow-up for multi-level cohorts. No significance was evident between cohorts for pre-operative cervical lordosis (p = 0.88). Similarly, no significance existed on post-operative measurements (SMD: 0.03; 95% CI -0.30,0.23; p = 0.63).

## 4 Discussion

Since its first description by Robinson and Smith, rates of anterior cervical surgery have been rising due to increased popularity of the surgical approach. Saifi et al. (2018) report that 1,059,043 ACDF procedures were performed in the United States between the years 2006–2013, a mean of 132,425 per year. Despite its popularity, there is still notable morbidity associated with ACDF surgeries (Fountas et al., 2007; Epstein, 2019). In a comprehensive review, Epstein et al. (Epstein, 2019) highlight overall morbidity rates of 13.2%–19.3%, with dysphagia, ASD, cerebrospinal fluid (CSF) leaks and postoperative haematomas representing procedure specific complications. Dysphagia is major concern due to its impact on the patient's quality of life in the postoperative period. A significant proportion of cases subside in the first few days after surgery due to the resolution of prevertebral swelling, however some cases of dysphagia persist for weeks or months and require SLT intervention (Fountas et al., 2007; Epstein, 2019).

ASD represents a major concern as it can result in revision surgery (Fountas et al., 2007; Epstein, 2019). The cervical spine is highly mobile in comparison to other spinal regions. During ACDF this region is stabilized, thus limiting motion, at the operative cervical segment. The mobility retained in adjacent superior and inferior segments can influence abnormal biomechanical loading patterns and ultimately the development of ASD. Limanówka et al. (Limanowka and Sagan, 2020) show that in a study of 28 patients who underwent ACDF for cervical spondylosis, patients who developed ASD superior to the fixation site had significantly greater extension range of motion (ROM) compared to those who did not develop ASD (p = 0.032). Such findings are corroborated by Park et al. (2007) in a cadaveric model of five cervical spine specimens. ACDF was performed with plate placement at C5-7, with intradiscal pressure and segmental motion recorded at C4-5 and C4-7 respectively. Mean intradiscal pressure was significantly higher post-ACDF (2475 ± 75 mmHg vs 1275 ± 225 mmHg; p < 0.05) and was associated with both increased flexion (3.74° ± 1.06° vs 2.27° ± 1.56°; change of 61%), increased extension (4.73° ± 2.27° vs 1.26° ± 1.42°; change of 275%) and lateral bending (3.37° ± 1.76° vs 2.46° ± 1.36°) at the C4-5 level (Park et al., 2007). To add to this base of knowledge, Chien et al. (2015) report in their study that this increase seen in ROM in the postoperative period typically resolves by three-months follow-up in single-level cases. However, it can last up-to and beyond 12-months in multi-level cases, contributing to the fact that multi-level ACDFs have been known to have higher rates of ASD than single-level cases (Epstein, 2019). Veeravagu report from their study of 92,867 patients with >24-months follow-up, that revision rates for single-level ACDF were 9.13% compared to 10.7% for multi-level procedures (OR: 1.2; 95% CI 1.1:1.3; p = 0.001), with multi-level cases more likely to have undergone revision surgery by 2-year follow-up (OR:1.1; 95% CI 1.0–1.2; p = 0.001) (Veeravagu et al., 2014). Revision surgery carries further risk of surgical complications and mortality, in addition to high associated cost, with revision surgeries for ACDF noted to cost approximately $17,514 (Bonano et al., 2022). “A potential contributing factor to ASD and ultimately ACDF revision surgery is the composition of cage and plate implants, traditionally made from titanium. Titanium and its alloys are known for their high strength-to-weight ratio, corrosion resistance, and stiffness. The modulus of elasticity for titanium ranges from approximately 100–110 GPa, depending on the specific alloy and processing (Arias-González et al., 2022). Comparatively, many commercial stand-alone spacers are composed of Polyetheretherketone (PEEK) coated with radiopaque titanium to facilitate visualisation on imaging. PEEK is a thermoplastic polymer known for its radiolucency, biocompatibility, and elasticity more closely resembling that of cortical bone. In comparison to metals like titanium, PEEK has lower stiffness or modulus of elasticity of approximately 3–4 GPa (Liao et al., 2020). Due to its lower modulus of elasticity, PEEK implants tend to exhibit more flexibility compared to metal implants. This flexibility can be advantageous in certain spinal applications, especially for implants designed to mimic the natural movement of the spine or reduce stress shielding effects on adjacent vertebrae, such as the case of the cervical spine. Therefore, PEEK implants might be preferred in cases where more flexibility or shock absorption is desired, such as in dynamic stabilisation systems or for patients with specific mobility requirements. Titanium implants might be favoured when higher stiffness and load-bearing capacity are needed, such as in lumbar fusion procedures or instances requiring greater structural support. Nevertheless, the stiffness of the implant should be compatible with the biomechanics of the treated spinal segment to promote successful fusion or motion preservation while minimizing stress on adjacent segments.

An additional, iatrogenic cause of revision surgery can result from annular tears caused during exposure of surgical field for cage and plate instrumentation. During ACDF surgery, complete removal of the entire disc is not typically necessary unless there are specific reasons, such as severe degeneration or structural issues, as determined by the surgeon during the procedure (Leven et al., 2017). Preserving some of the natural disc tissue helps maintain stability and function in the cervical spine while promoting successful fusion between the vertebrae. The bone graft or implant placed in the disc space serves to restore disc height, promote bone growth, and facilitate fusion between the adjacent vertebrae over time. A larger surgical approach, such as that used for cage and plate implants might necessitate more manipulation and retraction of tissues, potentially increasing the risk of unintended damage to the annulus fibrosus. Despite careful surgical techniques, a wider exposure increases the chances of accidental damage to adjacent structures, including the annulus fibrosus, especially when performing discectomy or preparing the vertebral endplates for fusion."

Therefore, surgeons have strived for improved surgical techniques and implants to mitigate aforementioned morbidity, such as stand-alone spacers in anterior cervical surgery. Several models exist on the current market and include Zero-P® (J&J, New Jersey, United States), Coalition® (Globus Medical, Pennsylvania, United States), HiJack® (Atlas Spine, Florida, United States), Lonestar® (OrthoFix, Texas, United States) and F3D-C2® (Corelink, Missouri, United States) among others. The majority of available stand-alone spacers have a similar functional design in which they are designed with a nodular superior and inferior surface to facilitate “grip” of adjacent vertebral bodies. This grip is complemented with obliquely-placed screws on the anterior aspect of the spacer, directed towards the middle of adjacent vertebral bodies (Nemoto et al., 2015; Yan and Nie, 2015; Chen et al., 2016; Liu et al., 2016; Li et al., 2017; Panchal et al., 2017; Xiao et al., 2017; He et al., 2018; Scholz et al., 2020). However, the comprehensive comparative design, outcomes and cost of commercially available stand-alone spacers is beyond the scope of this study.

Dysphagia is a significant driver of morbidity post-operatively in anterior cervical spine surgeries. Our study found that there was decreased dysphagia rates after 3 months when stand-alone spacers were used (27/284, 9.5% vs 49/291, 21.2%; RR: 0.59; p = 0.04). Interestingly, there was no significant difference at the 3 month follow-up (17/143, 11.9% vs 24/142, 16.9%; RR: 0.65; p = 0.62). While this study is unable to clarify why this is present, we suspect that dysphagia rates up until this point would be related to the surgical procedure. A previous systematic review performed by Cho et al., in 2013 explored dysphagia following anterior cervical spine surgery (Cho et al., 2013). Their findings showed that dysphagia was generally a multi-factorial process but identified multiple risk factors, including; female sex, revision surgery, more operative levels, longer operative time, older age, and the use of bulkier plates among others (Cho et al., 2013). Hardware characteristics such as size have been shown to predispose patients to prolonged dysphagia (Cho et al., 2013; Fisahn et al., 2018). Given these previous results we suspect that the persistent dysphagia rate longer than 3 months to be more related to hardware characteristics and less related to operative characteristics.

Our study results highlight reduced operative time (p = 0.05) alongside significantly reduced EBL (p < 0.01), total complications (p < 0.01), dysphagia rates (p = 0.04) and ASD rates (p = 0.04) for stand-alone spacers. However, is it important to note that although significant, average EBL was less that 30 ml and thus not clinically relevant. Furthermore, inability to accurately segregate single-level vs multi-level surgery in regards to operative time results in a large degree of heterogeneity, and results concerning operative should thus be inferred cautiously. Nevertheless, positive results complimented by similar radiological outcomes at follow-up in terms of restoration of spinal segment disc height and reduction of cervical lordosis, in addition to improved PROMs. Comparatively to CDA, in a meta-analysis by Gendreau et al. (2020) of 14 studies of which 4 were randomised prospective studies, CDA was shown to have reduced rates of ASD (OR: 0.56; 95% CI -0.06:1.18; p = 0.0745) dysphagia (OR: 0.32; 95% CI -0.21:0.84; p = 0.2368), operative time (SMD: 2.94 min; 95% CI -13.12:7.24; p = 0.5715), with no difference in NDI (SMD: 0.16; 95% CI -0.53:0.20; p = 0.3749) and VAS neck (SMD: 0.16; 95% CI -0.99:1.31; p = 0.7867) scores when compared to anchored spacers. However, as aforementioned, CDA cannot be employed in every instance, and further robust prospective studies of matched cohorts are needed for definitive comparison. Regardless, the results of this study highlight the benefits of employing stand-alone spacers in lieu of conventional cage and plate, and negate theoretical worries of insufficient instability with stand-alone spacers regardless of single-level of multi-level pathology.

Nevertheless, there are certain limitations to this study. The majority of studies concerned degenerative cohorts and thus our findings cannot be generalised to traumatic cohorts with significant instability or complex fracture patterns. Heterogeneity exists as certain studies did not outline at what time-point in the postoperative period clinical and radiological outcomes were evaluated. With respect to blood loss, though statistically significance is presence, it is unlikely that there is clinical significance as the blood loss difference is minimal. Importantly, there is some risk of industry bias as 2/10 studies had authors that are in financial relationships with the implant companies. Furthermore, implant heterogeneity can impact results. Additionally, further investigation is needed to define the extent to which stand-alone spacers can be used in anterior cervical fusion surgery. Certain studies have reported similar outcomes in that of 3-level fusion for stand-alone spacers versus cage and plate. Guo et al. report reduced incidence of dysphagia at 3-months and 6-months (p = 0.03) for stand-alone spacers (Guo et al., 2022). Nevertheless, this study was not randomised and therefore not included in this study. Several studies included in this study employed stand-alone spacers in 2-levels. Future robust pre-clinical and clinical studies should focus on biomechanical analysis and finite element modelling, with cyclic loading patterns to forecast the risk of long-term failure if used beyond 2-levels, with comprehensive analysis of revision surgeries with anchored spacers. However, our findings depict the efficiency of stand-alone spacers in the management of single- and multi-level cervical injury.

## 5 Conclusion

The results of this meta-analysis highlight the efficacy of stand-alone spacers for the management of primarily cervical spondylitic disease for both single-level and multi-level pathology, particularly in regards to EBL and associated complications, with similar PROMs and radiological outcomes. Thus, stand-alone spacers represent an attractive alternative to conventional instrumentation for patients undergoing ACDF surgery, particularly for spondylytic aetiology.

## Source of funding

None.

## Declaration of interests

The authors declare that they have no known competing financial interests or personal relationships that could have appeared to influence the work reported in this paper.

## Declaration of competing interest

None.
